# Morphological Study of the Anterior Surface of the Distal Radius

**DOI:** 10.1155/2017/8963768

**Published:** 2017-06-22

**Authors:** Ho-Jung Cho, Soyeon Kim, Dai-Soon Kwak

**Affiliations:** Department of Anatomy, Catholic Institute for Applied Anatomy, College of Medicine, The Catholic University of Korea, 222 Banpo-daero, Seocho-gu, Seoul 06591, Republic of Korea

## Abstract

The shape of the anterior surface of the distal radius is important for designing a distal radius plate for wrist fracture surgery. The aim of this study was to describe the shape of the anterior surface of the distal radius and to compare the results between female and male. We used 90 sides from three-dimensional radial models based on computer tomography images from Korean adult cadavers for this study. The anterior surface was measured in two dimensions in the coronal view, and we sectioned the anterior surface of the models to obtain intermediate and radial sections to measure the curved shape of the anterior surface in sagittal view. Several parameters were statistically different between females and males; however, there were no differences between the right and left sides for any parameters. The width of the anterior surface in the coronal view was larger in males than females, and the curved part of the anterior surface of the males was longer and more concave than that of females. In both the female and male specimens, the intermediate section was longer and more concave than the radial section. Our results are useful for anthropological studies and for designing distal radial plates.

## 1. Introduction

The radius is one of the long bones, and its distal part is broader than its proximal part. The shaft widens rapidly toward its distal end, and its distal part is anteriorly concave [1]. There have been several morphological studies of the radius in the fields of forensic anthropology and orthopedics. In forensic anthropology, several researchers have studied the radius to identify features useful for sex determination, specifically using the whole length and head diameter of the radius or the surface area and volume from three-dimensional (3D) radius models [2–8]. In orthopedic research, the shape of the distal radius has been studied for understanding of distal wrist fractures and plate design [9–15].

Recently, the frequency of wrist fractures has been rising. One reason is that there is an increasing number of elderly who fall, which is accompanied by a rise in the elderly population due to prolonged life expectancy. Another reason is that there are an increasing number of people who slip on icy streets in snowy weather, which is influenced by abnormal climate changes. Lastly, postmenopausal women have a higher risk of wrist fracture due to bone density loss. According to the National Health Insurance Sharing Service of Korea, the frequency of wrist and hand fractures increased by 2.5% from 2013 to 2014, while treatment costs increased by 29.6%. Therefore, it is necessary to review surgical methods for the distal part of the radius and describe the shape of the anterior surface to design proper plates and reduce complications after surgery. Some researchers have been studying the shape of the distal radius for developing and evaluating suitable plates for wrist surgery [11–15]; while some of these studies have shown the concave shape of the anterior surface of the distal radius at an angle with regard to the shape of plate, there is still lack of morphological information about the anterior surface of the distal radius.

The aim of this study was to describe and measure the concavity of the anterior surface of the distal radius and to compare the results between female and male. Furthermore, we suggest that the morphometric characteristics of the distal radius that we describe herein could be helpful for designing distal radial plates.

## 2. Materials and Methods

### 2.1. Materials

**Figure 1:**
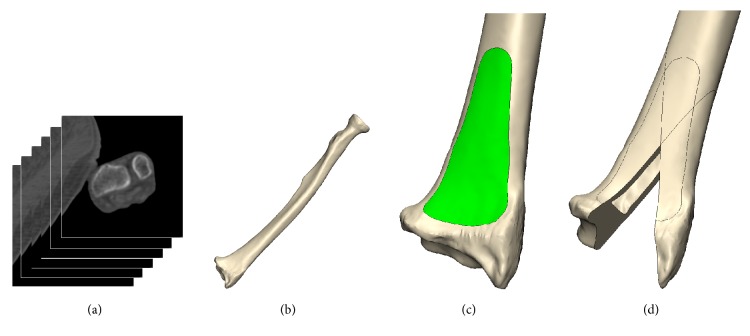
Process for reconstructing the three-dimensional (3D) radial model and the sectional model using a modeling program. (a) Obtained computer tomography (CT) images of the entire forearm, (b) 3D radial model reconstructed with the modeling program, (c) isolated anterior surface from the aligned 3D radial model, and (d) cutting 3D radial model to make the radial and intermediate sections.

For this study, we used 3D models of 90 sides of the radius that were made from computer tomography (CT) images from Korean adult cadavers. Thirty-four sides were obtained from females whose mean age was 79 years, and 56 sides were obtained from males whose mean age was 72 years. Whole forearms were scanned to 0.75 mm of slice thickness and 0.43 mm of pixel dimension to assure high quality of the CT images. Experts excluded radius that showed evidence of deformity, fracture, or surgical history on CT images. 3D radial models were made by 3D modeling program (Mimics, Materialise, Belgium) using CT images (Figure 1). To calibrate the 3D models, a plastic ball with a 2.25 inch diameter was scanned along with each radius, and the diameter of the real ball was compared with its 3D model, which was obtained from CT images.

### 2.2. Methods

The 3D radial models were aligned so that the anterior surface of the distal radius was visible for measuring the surface. The distal 25% of the whole radius length was used for alignment, in consideration of its influence on the curved shape of the radial shaft. The coronal plane was aligned so that the anterior flat surface of the distal radius was oriented toward the front side. The alignment of the sagittal plane was also established to be perpendicular to a straight line formed by the anterior flat surface of the distal 25% of the radius. The anterior surface was isolated from the aligned 3D distal radial models (Figure 1). The border of the anterior surface was defined based on morphological analysis of the surgical plate space. The boundary of the anterior surface was assessed by anatomical experts and confirmed by an orthopedist.

**Figure 2:**
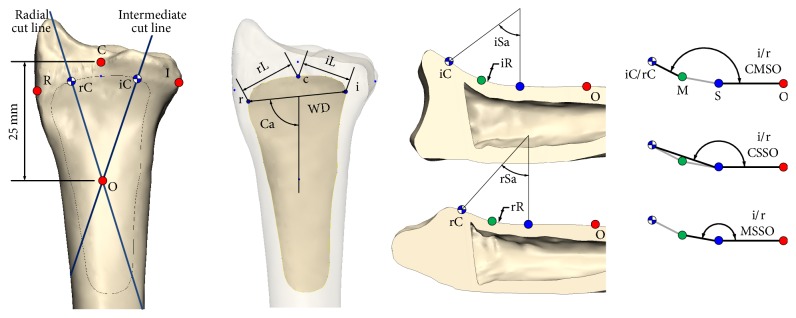
Landmarks and measurement methods for parameters.* Point C*: the point between the articular surface of the lunate and scaphoid at the carpal articular surface at the radial edge;* point I*: the most medial point of the edge of the distal radial model,* point R*: the most lateral point of the edge of the distal radial model;* point c*: the most distal edge of the separated anterior surface from the distal radial model;* point i*: the most medial point of the separated anterior surface from the distal radial model;* point r*: the most lateral point of the separated anterior surface from the distal radial model;* point O*: proximal area 25 mm from point C;* point S*: beginning point of the curvature from the sectioned distal radius;* point iC*: the end point of the curvature from the intermediate section;* point rC*: the end point of the curvature from the radial section;* point M*: the midpoint between the beginning point and the end point of the sectioned model.

Landmarks were established to measure several parameters (Figure 2), and our measurement methods are described in Table 1. We measured the separated anterior surface of the radius to describe the shape of the anterior surface of the coronal plane. We performed three different width measurements of the anterior surface: (1) the width of the intermediate and radial part of the distal edge of the anterior surface (iL and rL); (2) the width of the widest part of the anterior surface (WD); and (3) the inclined form of the anterior surface (Ca). Thereafter, the radial cut line, which passes through point O and the center of points R and C, and the intermediate cut line, which passes through point O and the center of points I and C, were made to describe the shape of the concave anterior surface. Using these two cut lines, the radial model was divided into two sections, the radial section and the intermediate section, and the concave shape of the distal radius was calculated with these sections in sagittal view. After sectioning the radial model, several points were fitted into each section, and then we calculated the equation for measuring a circle using the least-squares method using the fitted points, which expresses the curved shape of each section. The shape of the curved surface of the distal radius was described using several parameters (iSa, rSa, iR, and rR) of this circle. Lastly, the curved shapes of each section were calculated into angles using the landmarks for each section (iCMSO/rCMSO, iCSSO/rCSSO, and iMSSO/rMSSO).

All parameters were statistically analyzed, and we compared female and male measurements for the right and left sides using Student's *t*-test.

## 3. Results

Table 2 presents the results for all parameters. There were no significant differences between the right and left sides for any parameters, but some parameters showed statistical differences between females and males. For the separated anterior surface, the mean intermediate width (iL) was 12.04 ± 0.99 mm for females and 13.36 ± 1.40 mm for males, while the mean radial width (rL) was 12.03 ± 1.30 mm for females and 13.51 ± 1.10 mm for males; both iL (*p* < 0.01) and rL (*p* < 0.01) were statistically different between females and males. For the total population, the mean iL was 12.85 ± 1.41 mm, and the mean rL was 12.94 ± 1.38 mm; these measurements were not significantly different. The mean anterior surface width (WD) was 20.82 ± 1.42 mm for females and 23.44 ± 1.39 mm for males. The WD for males was significantly broader than for females (*p* < 0.01). The mean inclined angle of the anterior surface (Ca) was 87.68 ± 5.50° for females, 86.59 ± 3.85° for males, and 87.01 ± 4.56° for the total sample; the difference between females and males was not significant.

We defined the curved shape of the anterior surface of the intermediate and radial sections as the arc of the circle in sagittal view. The mean angle of curvature of the intermediate section (iSa) was 32.64 ± 11.76° for females and 38.25 ± 12.45° for males, and the mean angle of curvature of the radial section (rSa) was 26.04 ± 10.43° for females and 31.79 ± 11.15° for males. The curved shape of the anterior section for males was longer than that for females, but the difference was not significant. For the total sample, the mean iSa was 36.10 ± 12.43°, and the mean rSa was 29.56 ± 11.17°; therefore, the curved shape of the intermediate anterior section was longer than the radial section (*p* < 0.01). The mean radius of curvature of the intermediate section (iR) was 24.89 ± 9.50 mm for females and 22.63 ± 9.03 mm for males, and the mean radius of curvature of the radial section (rR) was 29.23 ± 11.76 mm for females and 25.97 ± 12.13 mm for males. The anterior surface of both sections was more concave among males than females, but not significantly so.

The curved shape of the intermediate and radial sections was articulated into angles using landmarks located at the starting point, midpoint, and end point of the curved part of the anterior surface. The mean angle between the flat surface and the line connecting the starting point and midpoint (iMSSO and rMSSO) and the mean angle between the flat surface and the line connecting the midpoint and end point (iCMSO and rCMSO) were larger among females than males. Generally, the angle of the anterior surface tended to be larger in females than males (*p* < 0.01). The radial section measurements were larger than those of the intermediate section in both groups; accordingly, the intermediate section was more concave than the radial section (*p* < 0.02). The mean angle between the flat surface and the line connecting the starting point and end point (iCSSO and rCSSO) was greater in females than males for both sections (*p* < 0.01 for both). The mean iCSSO and rCSSO were 161.62 ± 4.99° and 164.13 ± 4.41°, respectively, for females and males. The angle of the radial section was larger than that of the intermediate section (*p* < 0.01).

## 4. Discussion

The distal end of the radius is wide, and its anterior surface has a concave shape. This anterior surface is clinically important because the distal radius plate should be well placed on the anterior surface during distal radius surgery. However, there was a lack of anatomical studies that specifically analyze the anterior surface of the distal radius. Therefore, we measured and analyzed the morphological characteristics of the anterior surface in relation to the radial plate to aid in surgical planning. In previous studies, the angle of the concave anterior surface was measured using real dry bones or medical images in order to analyze the distal radial shape [11, 12, 15]. However, we used 3D radial bone models and measured the anterior surface in the front and side views of the sectioned model.

In this study, we tried to define the shape of the anterior surface of the distal radius in Koreans. When we measured the width of the anterior surface by separating it into intermediate and radial parts, the radial width (rL) was bigger than the intermediate width (iL), although the difference was not significant. However, when we compared the iL, rL, and the whole width (WD) between females and males, all measurements were smaller in females than males (*p* < 0.01). The curved section of the anterior surface of the intermediate section was longer and more concave than the radial section. The curved shape for males tended to be longer and more concave than for females.

We compared the shape of the curved section with previous studies' measurements using several parameters. Gasse et al. [11] used dry radii, which were collected from two universities in France, to measure the angle between the flat surface and the line connecting the starting point and end point (CSSO) at the lateral and intermediate sections. The average angle of the CSSO was 144.9° for the intermediate section and 155.3° for the lateral section; thus, the curved shape of the anterior surface from Gasse et al.'s measurements was more concave than in our results. We found a difference of about 3° between the intermediate and lateral sections (iCSSO = 161.62° and rCSSO = 164.13°), while in Gasse et al.'s study, the difference between iCSSO and rCSSO was 10°. We assume that the discrepancies between the studies were due to different sectioning standards. In their study of a UK population, Evans et al. [12] estimated 149° for the lateral section, 164.2° for the middle section, and 145.9° for the medial section. Similar to Gasse et al.'s study, Evans et al. found that the shape of the anterior surface was more concave, which contrasts with our study. Oppermann et al. [15] measured the angle between the flat surface and the line connecting the midpoint and end point (CMSO) to analyze the anterior surface angle in a German population; they found that the angle of the lateral section was 159.37° for females, 153.60° for males, and 156.07 for the total sample. The angle of the medial section was 149.41° for females, 147.38° for males, and 148.25° for the total sample. Similar to our findings, they found that the angles were greater in the female subjects than the male subjects. Their measurement for the angle of the lateral section was also consistent with our study; however, their measurement for the angle of the medial section was smaller than ours. Other than the angles for CSSO and CMSO, there were no other parameters that we could compare between the studies.

The results of this study are helpful for designing a distal radius plate. The intermediate and radial widths are useful for deciding the best head size of a double-column plate, and the whole width can be used to define the head size of a single-column plate. To design a plate that is well fitted to the bone surface, the curve of the anterior surface should be properly reflected. We calculated the fitted circle from the anterior surface using a sectioned model and the least-squares method. We measured several parameters, including iSa, rSa, iR, and rR, using this circle. We suggest that these parameters could be useful for designing the curve shape of surgical plates. Lastly, the results of the angle of the anterior surface of the sagittal section (CMSO, CSSO, and MSSO) may contribute to preparation of plates with a flat head.

This study has several limitations. First, we used only Korean radii for our measurements. Several previous authors from other countries investigated the shape of the anterior surface of the distal radius using the angle between the flat surface and the line with a curved shape [11, 12, 15], and some of the parameters that we used were measured using a similar method; however, we could not precisely compare results because the sectioning methods differed between the studies. We presume that our sectioning method is more appropriate than previous methods for designing detailed radial plates, particularly double-column plates. Therefore, we used our sectioning method to increase the utilization of our results from this study. Second, we measured only the anterior surface of the distal part of the radius, not the whole radius. There are no meaningful characteristics of the proximal or body parts of the radius, except the rounded head. Furthermore, the whole length and head diameter of the radius have been well studied with regard to sexual dimorphism. However, there is a lack of morphological study of the anterior surface of the distal radius.

## 5. Conclusion

This study investigated the shape of the anterior surface of the distal radius. The width of the anterior surface was greater in males than females. When we compared the measurements for the sectioned radius, the curved section of the intermediate side was longer and more curved than the radial side, and male shapes tended to be more concave than female shapes. We could not directly compare our results with those from previous studies, but the anterior surface of the Korean radius, according to our results, had a flatter surface than those from other countries. Our results are helpful for anthropological study of the distal radius and for orthopedics research into designing distal radial plates for fracture surgery.

## Figures and Tables

**Table 1:** Measurement methods for distal radius parameters.

Regions	Abbreviation	Measurement
Anterior surface in coronal view	iL	Intermediate width/length between points i and c
	rL	Radial width/length between points r and c
	WD	Whole width/length between points i and r
	Ca	Angle between the connected line using points i and r and the central vertical line of the radius

Curvatures of the anterior surface in sagittal sectional view	iSa	Angle of curvature of the curved surface of the anterior surface as an arc on the intermediate section
	rSa	Angle of curvature of the curved surface of the anterior surface as an arc on the radial section
	iR	Radius of curvature of the curved surface of the anterior surface on the intermediate section
	rR	Radius of curvature of the curved surface of the anterior surface on the radial section

Angles of the anterior surface in sagittal sectional view	iCMSO	Angle of the line connecting points iC and M and the line connecting points S and O
	rCMSO	Angle of the line connecting points rC and M and the line connecting points S and O
	iCSSO	Angle of the line connecting points iC and S and the line connecting points S and O
	rCSSO	Angle of the line connecting points rC and S and the line connecting points S and O
	iMSSO	Angle of the line connecting points iM and S and the line connecting points S and O
	rMSSO	Angle of the line connecting points rM and S and the line connecting points S and O

**Table 2:** Results of the distal radius parameters.

Parameters	Female		Male		Combined	
	Mean	SD	Mean	S.D.	Mean	SD
iL^†^ [mm]	12.04	0.99	13.36	1.40	12.85	1.41

rL^†^ [mm]	12.03	1.30	13.51	1.10	12.94	1.38

WD^†^ [mm]	20.82	1.42	23.44	1.39	22.42	1.90

Ca [°]	87.68	5.50	86.59	3.85	87.01	4.56

iSa [°]	32.64	11.76	38.25	12.45	36.10	12.43
rSa [°]	26.04	10.43	31.79	11.15	29.56	11.17

iR [mm]	24.89	9.50	22.63	9.03	23.50	9.22
rR [mm]	29.23	11.76	25.97	12.13	27.21	12.03

iCMSO^†^ [°]	154.80	6.03	150.12	7.95	151.92	7.59
rCMSO^†^ [°]	159.48	6.81	153.91	6.61	156.07	7.19

iCSSO^†^ [°]	163.91	3.90	160.19	5.10	161.62	4.99
rCSSO^†^ [°]	166.45	4.05	162.66	4.01	164.13	4.41

iMSSO^†^ [°]	170.90	2.24	169.22	2.58	169.87	2.58
rMSSO^†^ [°]	172.49	2.81	169.81	2.87	170.85	3.12

^†^ *p* < 0.01.
